# Age of onset in familial breast cancer as background data for medical surveillance

**DOI:** 10.1038/sj.bjc.6605421

**Published:** 2009-11-10

**Authors:** A Brandt, J Lorenzo Bermejo, J Sundquist, K Hemminki

**Affiliations:** 1 Division of Molecular Genetic Epidemiology, German Cancer Research Centre (DKFZ), 69120 Heidelberg, Germany; 2 Institute of Medical Biometry and Informatics, University Hospital Heidelberg, 69120 Heidelberg, Germany; 3 Center for Primary Care Research, Lund University, 205 02 Malmö, Sweden; 4 Stanford Prevention Research Center, Stanford University School of Medicine, Stanford, California 5411, USA

**Keywords:** breast cancer, familial breast cancer, age of onset, screening recommendations, cumulative risk

## Abstract

**Background:**

Familial breast cancers are known to be of early onset. This article provides differences in the age of onset of breast cancer and death by breast cancer between women with and without a family history.

**Methods:**

The Swedish Family-Cancer Database was used to estimate the cumulative risk of breast cancer and death by breast cancer according to family history with a stratified Cox model. Family history was defined separately for affected mother or sister considering their diagnostic ages.

**Results:**

The age to reach the same cumulative incidence as women without family history decreased with decreasing diagnostic age of the affected relative. Women with a maternal history reached the risk of women lacking a family history at the age of 50 years between 12.3 (mother affected <40 years) and 3.3 years (mother affected >82 years) earlier. The trend for breast cancer mortality was essentially similar.

**Conclusions:**

Women with mother or sister affected by breast cancer are diagnosed and die at earlier ages than do women without family history. The differences depend on the diagnostic age of the affected relative. The present data may provide a rationale to derive recommendations for the starting age of screening in women with affected family members.

A family history of female breast cancer is associated with an increase in the risk of breast cancer in first-degree female relatives by about two-fold, but the magnitude of risk depends on a number of factors, such as diagnostic age (Collaborative Group on Hormonal Factors in Breast Cancer, 2001; Hemminki *et al*, 2008b). Familial risk has been included in clinical risk estimation models for breast cancer (Gail *et al*, 1989; Claus *et al*, 1994; Tyrer *et al*, 2004), but the manner in which it could be translated into recommendations for a surveillance strategy for at-risk women requires scientific justification (Smith *et al*, 2004, 2006; Saslow *et al*, 2007). The guidelines for breast cancer screening of average-risk individuals were based on trials investigating mortality reduction by cancer screening, and the starting age was determined by the onset of breast cancer incidence (IARC, 2002). Although familial breast cancers are known to be of early onset (Claus *et al*, 1990; Collaborative Group on Hormonal Factors in Breast Cancer, 2001; Hemminki *et al*, 2008b), data have not been accurate enough to provide a scientific basis for the existing recommendations for the time of implementation of screening methods (Smith *et al*, 2004). The recommendations for at-risk women emphasise breast cancer diagnosis in relatives before the age of 50 years, thereby leaving open the question about breast cancer in older women.

The aim of this study was to assess (1) the cumulative incidence and risk of death from breast cancer in women with a family history of breast cancer compared with those without a family history of breast cancer and (2) the impact of the relative's age at diagnosis on the diagnostic age. We used the nation-wide Swedish Family-Cancer Database to estimate the cumulative incidence of breast cancer and the cumulative risk of death by breast cancer in women with a family history of breast cancer, compared with those lacking a family history. Family history was defined separately for an affected mother or sister considering their diagnostic ages. The results may encourage appropriate future analysis to derive recommendations for the starting time of screening in women with an affected family member.

## Materials and methods

The Swedish Family-Cancer Database was created in the 1990s by linking information from the Multigeneration Register, national censuses, Swedish Cancer Registry and death notifications (Hemminki *et al*, 2001). Data on family relationships were obtained from the Multigeneration Register, in which all individuals born in or after 1932 are registered with their biological parents as families; in addition, data on immigrants were included. Thus, the individuals in the Database can be divided into offspring generation (individuals born in or after 1932) and parental generation. The nation-wide Swedish Cancer Registry was established in 1958 and reached complete coverage in 1961. It is based on compulsory reports of diagnosed cases, with coverage of cancer registration close to 100% (Centre for Epidemiology, 2005). The 2006 update of the Database includes more than 11.5 million individuals and cancer cases from years 1958 to 2004 and the underlying cause of death until 2003 (Hemminki *et al*, 2006). Further details on the Database are described elsewhere (Hemminki *et al*, 2001, 2006). The cancers in the Database are coded according to the 7th International Classification of Diseases (ICD-7). Breast cancer in this study refers to first primary invasive breast cancer (ICD-7 code 170). The study population consisted of women from the offspring generation with two identified parents, in total 3.6 million women. Women without identified parents, who were mostly immigrants, were excluded from the study. The age structure of the Database (children born after 1932) implicates that the maximum age of diagnosis of affected sisters is 72 years; the age of mothers was not limited.

Women with mother or sister affected by breast cancer were defined to be at familial risk. Here, the register-based definition of family history was used, that is, family history was defined independently of the maternal or sororal diagnostic date. Cumulative risks of breast cancer and death due to breast cancer according to family history were estimated using a stratified Cox model based on Tsiatis' method (Tsiatis, 1981) (PROC PHREG; SAS version 9.1; SAS Institute, Cary, NC, USA). The strata were defined according to the disease status of the mother or sister and according to their diagnostic ages. Women with multiple affected relatives were analysed separately. Individuals entered the risk period at birth, at immigration date, or at first year of the study (1961). Immigrants might have had breast cancer diagnosed in their country of origin; however, the median age at immigration for immigrants in the Database was below 30 years. For analysis of the age of diagnosis with breast cancer, the censoring events were death, emigration, 31 December 31 2004, absence at census and diagnosis of malignancy at sites other than breast. Women were also censored at diagnosis of malignancy at sites other than breast, because their risk afterwards might be different from the risk in the general population. For the analysis of the age of death from breast cancer, censoring events were the same, but ‘death’ was replaced by ‘death from another cause than breast cancer’. Socioeconomic status, calendar period, age at first birth, number of children and region were taken into account as covariates. Socioeconomic status was based on the Swedish socioeconomic classification, which relies primarily on occupation (Statistics Sweden, 1982). In this study, categories ‘Farmer’, ‘Blue collar worker’, ‘White collar worker’, ‘Private’, ‘Professional’ and ‘Other/unknown’ were used. The categories of regions were ‘Big cities’, ‘South Sweden’, ‘North Sweden’ and ‘Unknown’. This categorisation was chosen because screening behaviour might differ between residents of these regions. For example, the proportion of women taking part in screening might be lower in North Sweden because of longer distances to the nearest screening centre. The socioeconomic index and region were determined at the event time or censoring time, respectively. We calculated the age at which the cumulative risks for women with an affected mother or sister reached the cumulative risk of women without a family history at the age of 40 or 50 years (Lai and Su, 2006).

## Results

Table 1 presents the number and median age according to family history for all women from the study population, for women diagnosed with breast cancer and for women who died from breast cancer.

**Figure 1:**
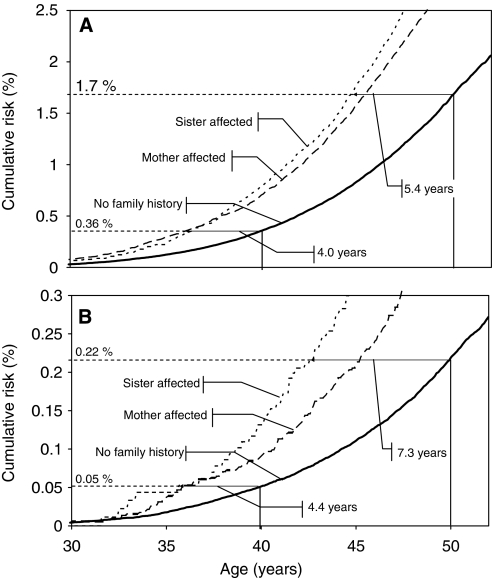
Cumulative incidence of breast cancer and cumulative risk of death by breast cancer according to the type of family history. (**A**) Age at which women with a family history reach the cumulative risk of women lacking a family history at the age of 40 and 50 years for incidence. (**B**) Age at which women with a family history reach the cumulative risk of women lacking a family history at the age of 40 and 50 years for death from breast cancer.

Figure 1 shows the cumulative incidence of breast cancer (Figure 1A) and the cumulative risk of death by breast cancer (Figure 1B) according to the type of family history (no family history, mother affected, sister affected). The cumulative incidence of breast cancer was almost equally high for women with an affected mother or sister; the familial incidence was almost two times higher than the incidence for women lacking a family history. We marked in Figure 1A the cumulative incidence (1.7%) reached by women lacking a family history at the age of 50 years. Women with a sororal history reached this cumulative incidence 5.4 years earlier; women with a maternal history reached this incidence 4.7 years earlier. At the age of 40 years, the cumulative incidence of women lacking a family history was 0.36%. This incidence was reached 3.7 years earlier by women with a sororal history and 4.0 years earlier by women with a maternal history. In Figure 1B, the cumulative risk of breast cancer death is shown. For women lacking a family history, mortality at the age of 40 years was 0.05% and that at the age of 50 years was 0.22%. These mortalities were reached 4.4 years and 7.3 years earlier, respectively, by women with a sororal history. Women with a maternal history reached these mortalities 4.2 years and 4.8 years earlier, respectively.

Table 2 (top part) shows the ages at which women with a maternal or sororal history of breast cancer reach the cumulative incidence of 1.7% for breast cancer, that is, the risk that women without a family history reached by the age of 50 years (see Figure 1), according to the diagnostic age of the affected family member. The age difference at diagnosis due to family history is shown by ‘AD’, which decreased from 12.3 years when the mother was diagnosed at age below 40 years to 3.3 years when she was diagnosed at age over 82 years. The age difference for sisters decreased from 8.9 years (sister diagnosed before the age of 40 years) to 3.0 years (sister diagnosed between 60 and 72 years). Data for age differences in mortality are shown in Table 2 (bottom part). There were few fatal events but the trend was essentially similar to the incidence data: diagnosis at an early age in one family member also predicted an early death of the second family member.

Table 3 shows the ages at which women with a maternal or sororal history of breast cancer reach the cumulative incidence of 0.36% for breast cancer, that is, the risk that women without a family history reached by the age 40 of years (see Figure 1), according to the diagnostic age of the affected family member. The age difference at diagnosis due to family history decreased from 9.5 years when the mother was diagnosed at an age below 40 years to 0.8 years when she was diagnosed at an age over 82 years. The age difference for sisters decreased from 7.0 years (sister diagnosed before the age of 40 years) to 0.9 years (sister diagnosed between 60 and 72 years). Fatal events were too few for a detailed analysis.

Women with an affected mother and sister, and women with more than one affected sister were considered separately (data not shown). The numbers of women affected by breast cancer in these groups were small. For women with a mother and a sister affected, the ages to reach the same risk as the general population at the age of 50 and 40 years were 41.3 years (33 cases until this age, 95% confidence interval (CI): 39.2–43.1 years) and 32.8 years (7 cases, 95% CI: 31.4–36.3 years), respectively. The corresponding ages for women with two affected sisters were 42.3 years (11 cases, 95% CI: 33.4–44.9 years) and 25.8 years (2 cases, 95% CI: 23.3–38.1 years), respectively. The numbers of deaths in women with a mother and a sister affected (27) or with two affected sisters (12) were too small for a separate analysis.

## Discussion

Our results provide initial data on the age of onset of familial cancer estimated with a large population-based data set. For example, if the general starting age of mammography screening is defined to be 50 years, our data show that the same cumulative risk is reached 9–12 years earlier for women with a mother or a sister affected by breast cancer before 40 years. According to previous recommendations, an earlier start of screening is not recommended for women with mother or sister who was affected older than 50 years. However, the present results indicated that these women are at an increased risk. The estimation of the age at which familial cases reached the risk of death in women without a family history permitted essentially identical conclusions, even though the results were based on a smaller numbers of events.

The American Cancer Society recommends average-risk women begin mammography screening at the age of 40 years (Smith *et al*, 2007). In the European Union, the Advisory Committee on Cancer Prevention recommends the introduction of organised mammography screening beginning at 50 years (Advisory Committee on Cancer Prevention, 2000). In most nationally organised programmes in Europe and Canada, the starting age of mammography screening is 50 years (IARC, 2002). These screening programmes do not take the family history of breast cancer into consideration, although some guidelines recommend individual strategies when risk factors are present (Albert and Schulz, 2004). The National Board of Health and Welfare recommends Swedish counties to offer mammography screening for women at the age of 40–74 years. There are differences between counties when they start screening and the screening interval (Olsson *et al*, 2000). The American Cancer Society recommends MRI (magnetic resonance imaging) screening as an adjunct to mammography screening for women with an estimated 20–25% or greater lifetime risk of breast cancer (Saslow *et al*, 2007). However, the starting age of screening for breast cancer in women at increased risk is not well established. The National Center for Clinical Excellence in the United Kingdom recommends annual mammography screening for at-risk women beginning at the age of 40 years, which is 10 years earlier than the recommended starting age in the United Kingdom for the general population (McIntosh *et al*, 2004); the increased risk is defined as an estimated lifetime risk of breast cancer between 17–30%, which includes women with a first-degree relative affected by breast cancer before 50 years and women with two affected first- or second-degree relatives (McIntosh *et al*, 2004). The American Cancer Society recommends women with a relative affected by breast cancer before 50 years, with two or more relatives affected by breast cancer and with a relative affected by two independent breast cancers to start 10 years earlier than average-risk women, or 5–10 years earlier than the youngest patient in the family (Smith *et al*, 2003).

The rationale of mammography screening is the reduction of mortality by breast cancer. As evidence of an impact of family history on the survival of breast cancer patients in general is lacking (Chappuis *et al*, 1999; Hemminki *et al*, 2008a), the cumulative risk of death by familial breast cancer is increased because of the increased incidence. The difference in the age of onset of familial cases might be influenced by overdiagnosis or an earlier detection of familial cases because of increased surveillance of familial cases: women with a close relative affected by breast cancer may participate in cancer screening more often, more frequently or earlier than women in the general population (Shah *et al*, 2007). The likelihood of lead-time bias motivated us to investigate the difference in breast cancer-specific mortality between women with and without affected relatives. The present data showed essentially the same differences in age of diagnosis and age of death between women with and without affected relatives. Thus, lead-time bias does not seem to influence the difference in the onset age of breast cancer in familial and non-familial cases. This is in agreement with earlier results that showed at most a minor influence of screening (Bermejo and Hemminki, 2005; Hemminki and Bermejo, 2005).

This study includes the whole Swedish population up to the age of 72 years and their parents. The information on cancer and diagnostic ages was registered data. Thus, an important advantage was the accuracy and completeness of the analysed data, which minimised biases related to over- and under-reporting of family history, selection and recall. The existing recommendations for surveillance of women at increased risk are mainly based on expert opinion, with support from the assessment of breast cancer risk with statistical models (Gail *et al*, 1989; Claus *et al*, 1994; Tyrer *et al*, 2004) or epidemiological studies (Smith *et al*, 2003; McIntosh *et al*, 2004). Different models for the prediction of breast cancer risk have been developed. These models include different combinations of risk factors. In the past, the most widely used models were the Gail model and the Claus model (Gail *et al*, 1989; Claus *et al*, 1994). The Gail model takes the number of affected first-degree relatives, age at menarche, age at first birth and the number of breast biopsies into account (Gail *et al*, 1989). This model does not consider the age at diagnosis and includes only first-degree relatives (Evans and Howell, 2007). The Claus model predicts lifetime risk of breast cancer for different combinations of affected first- and second-degree relatives (Claus *et al*, 1994). However, it does not include risk factors other than family history and it reflects the risk of breast cancer for women in the United States in the 1980s (Evans and Howell, 2007). The BRCAPRO model predicts breast cancer risk on the basis of the probability of carrying a mutation in *BRCA1*/*2*, taking cancer status and age of first- and second-degree relatives into account (Parmigiani *et al*, 1998). This model includes only *BRCA1*/*2* as genetic elements (Evans and Howell, 2007). The Breast and Ovarian Analysis of Disease Incidence and Carrier Estimation Algorithm (BOADICEA) model includes a polygenic component for breast cancer susceptibility in addition to *BRCA1*/*2* (Antoniou *et al*, 2004). The Tyrer–Cuzick model combines the prediction of genetic risk based on family history information and personal risk factors (Tyrer *et al*, 2004). This study presents empirical risk estimates on the age of onset based on breast cancers in first-degree relatives taking the relative's diagnosis age into account. Second-degree relatives were not considered because the structure of the Swedish Family-Cancer Database implies that identification of grandparents and aunts is only possible for women whose parents were born in 1932 or later, that is, only for women who were aged ∼50 years or less at the end of the study.

The efficacy of screening is not equivalent to case detection. There is ample evidence that the efficacy of mammography screening in average-risk women is lower in women aged 40–49 years than in those aged 50–69 years (IARC, 2002; Moss *et al*, 2006). No evidence has been found for the efficacy of screening under the age of 40 years. However, it has been shown that women with an increased genetic risk of breast cancer might benefit from an intensified surveillance, which includes an earlier start of mammography screening, expert clinical breast examination and teaching of ‘breast awareness’ (Moller *et al*, 1999). Further research is required to clarify the efficacy of breast cancer screening before 50 and 40 years in women at increased risk of breast cancer.

We conclude that women with mothers or sisters affected by breast cancer were diagnosed at earlier ages than were individuals without a family history. The differences in age of onset depended on the age of affected relatives, whereas the type of proband (mother or sister) seemed to have a minor function. Under the discussed limitations, the present data should encourage further analysis in order to derive evidence-based recommendations for the starting age of screening in women with a family history of breast cancer.

## Figures and Tables

**Table 1:** Number and median age of women in the whole study population, women diagnosed with breast cancer and women died from breast cancer according to family history

	**Study population**		**Women diagnosed with breast cancer**		**Women died from breast cancer**	
**Family history**	**No.**	**Median age at censoring or diagnosis (years)**	**No.**	**Median age at diagnosis (years)**	**No.**	**Median age at death (years)**
No family history	3 480 260	32	36 835	51	4793	53
						
*Mother affected*	120 108	46	3649	50	452	51
0–39 years	6428	27	88	39	20	42
40–49 years	23 168	34	381	45	42	47
50–59 years	31 728	40	715	48	81	49
60–72 years	37 029	49	1316	50	164	52
73–82 years	16 360	57	829	51	109	53
>82 years	5395	60	320	54	36	55
						
*Sister affected*	31 000	57	1780	52	228	52
0–39 years	3331	48	140	46	23	43
40–49 years	10 228	54	534	51	71	51
50–59 years	12 138	58	760	52	99	53
60–72 years	5303	61	346	54	35	56
						
Sister+mother affected	2794	56	252	49	27	53
						
Two sisters affected	925	58	76	51	12	55

**Table 2:** Age at which women with a family history reach the cumulative risk of women lacking a family history at age 50 years for incidence (top) and for death (bottom) considering the diagnostic age of the relative

	**Maternal history**					**Sororal history**				
**Diagnostic age of relative (years)**	**No.^a^**	**Age^b^ (years)**	**95% CI**		**AD^c^**	**No.^a^**	**Age^b^ (years)**	**95% CI**		**AD^c^**
*First breast cancer*										
0–39	36	37.7	36.9	40.0	−12.3	38	41.1	38.4	43.5	−8.9
40–49	158	43.3	42.2	44.2	−6.7	126	44.5	43.6	45.7	−5.5
50–59	264	44.7	43.8	45.6	−5.3	146	44.8	44.1	46.1	−5.2
60–72	429	46.0	45.4	46.8	−4.0	62	47.0	45.6	48.9	−3.0
73–82	214	46.1	45.2	46.9	−3.9					
>82	66	46.7	45.3	48.0	−3.3					
										
*Death by breast cancer*										
0–39	6	35.4	34.3	40.8	−14.6	9	38.3	32.5	42.6	−11.7
40–49	29	45.3	41.8	47.8	−4.7	31	44.5	42.3	46.8	−5.5
50–59	49	45.0	42.8	46.8	−5.0	32	43.9	39.4	47.4	−6.1
60–72	81	45.3	43.5	48.2	−4.7	12	49.7	37.4	56.3	−0.3
73–82	48	45.3	41.8	47.5	−4.7					
>82	6	42.8	29.8	52.7	−7.2					

Abbreviations: AD=age difference; CI=confidence interval.

a Number of cases until (Age).

b Age to reach the same cumulative risk as women lacking a family history at age 50.

c Difference between and ‘Age’ and 50 years.

**Table 3:** Age at which women with a family history reach the cumulative risk of women lacking a family history at age 40 years for incidence considering the diagnostic age of the relative

	**Maternal history**					**Sororal history**				
**Diagnostic age of relative (years)**	**No.^a^**	**Age^b^ (years)**	**95% CI**		**AD^c^**	**No.^a^**	**Age^b^ (years)**	**95% CI**		**AD^c^**
0–39	10	30.5	28.2	33.5	−9.5	8	33.0	28.4	35.1	−7.0
40–49	45	33.7	33.1	35.1	−6.3	24	35.2	33.6	37.7	−4.8
50–59	75	35.4	34.2	36.8	−4.6	28	38.1	36.5	39.0	−1.9
60–72	97	37.3	36.4	38.1	−2.8	11	39.1	37.1	41.8	−0.9
73–82	41	38.6	36.5	40.1	−1.4					
>82	12	39.3	35.3	41.2	−0.8					

Abbreviations: AD=age difference; CI=confidence interval.

a Number of cases until (Age).

b Age to reach the same cumulative risk as women lacking a family history at age 40.

c Difference between ‘Age’ and 40 years.
